# Bridging Drought Experiment and Modeling: Representing the Differential Sensitivities of Leaf Gas Exchange to Drought

**DOI:** 10.3389/fpls.2018.01965

**Published:** 2019-01-15

**Authors:** Shuang-Xi Zhou, I. Colin Prentice, Belinda E. Medlyn

**Affiliations:** ^1^Department of Biological Sciences, Macquarie University, Sydney, NSW, Australia; ^2^The New Zealand Institute for Plant and Food Research Ltd., Hawke’s Bay, New Zealand; ^3^AXA Chair of Biosphere and Climate Impacts, Grand Challenges in Ecosystems and the Environment and Grantham Institute – Climate Change and the Environment, Department of Life Sciences, Imperial College London, Ascot, United Kingdom; ^4^Hawkesbury Institute for the Environment, Western Sydney University, Penrith, NSW, Australia

**Keywords:** photosynthesis, stomatal and non-stomatal limitation, mesophyll conductance, *V*_cmax_, *J*_max_, drought acclimation, flux measurement, land surface model

## Abstract

Global climate change is expected to increase drought duration and intensity in certain regions while increasing rainfall in others. The quantitative consequences of increased drought for ecosystems are not easy to predict. Process-based models must be informed by experiments to determine the resilience of plants and ecosystems from different climates. Here, we demonstrate what and how experimentally derived quantitative information can improve the representation of stomatal and non-stomatal photosynthetic responses to drought in large-scale vegetation models. In particular, we review literature on the answers to four key questions: (1) Which photosynthetic processes are affected under short-term drought? (2) How do the stomatal and non-stomatal responses to short-term drought vary among species originating from different hydro-climates? (3) Do plants acclimate to prolonged water stress, and do mesic and xeric species differ in their degree of acclimation? (4) Does inclusion of experimentally based plant functional type specific stomatal and non-stomatal response functions to drought help Land Surface Models to reproduce key features of ecosystem responses to drought? We highlighted the need for evaluating model representations of the fundamental eco-physiological processes under drought. Taking differential drought sensitivity of different vegetation into account is necessary for Land Surface Models to accurately model drought responses, or the drought impacts on vegetation in drier environments may be over-estimated.

## Introduction

Soil water deficit is the main environmental driver that limits aboveground net primary production in land vegetation (Webb et al., 1983; Zeppel et al., 2014), and induces vegetation mortality on all six vegetated continents and for most biomes across the globe (Potts, 2003; Allen et al., 2010; Phillips et al., 2010; Anderegg et al., 2012; Choat et al., 2012; Williams et al., 2013). By affecting physiological (e.g., leaf gas exchange, canopy conductance), structural (e.g., leaf area, root length, mass distribution) and biogeographic (e.g., forest composition and species distribution) processes at the plant and community levels, extreme drought is expected to cause regional losses of biodiversity and biomass (Phillips et al., 2009) with impacts on ecosystem function and the terrestrial carbon sink (Pitman, 2003; Bonan, 2008; Phillips et al., 2010).

Modeling the quantitative consequences of increased drought for forest ecosystems is challenging (McDowell et al., 2011), and requires unraveling the interaction between drought and plant gas exchange at different time scales, and in different ecosystems with different degrees of adaptation to drought. Reliable model prediction of drought impacts on forest ecosystems must be based on the analysis of observations to identify key traits that promote plant resistance to drought, and process-based modeling to include realistic representation of the ecophysiological mechanisms relating plant gas exchange to water availability and transport. In addition, we need to understand how drought impacts vary among ecosystems. The drought impacts on different ecosystems depend on the drought duration, magnitude, and spatial extent, vegetation type-specific responses to drought at different time scales, and mechanisms affecting the drought resistance and resilience of different vegetation types (Pasho et al., 2011; Vicente-Serrano et al., 2013). Different terrestrial ecosystems are reported to differ in their sensitivity to drought (Knapp and Smith, 2001; Zeppel et al., 2014). For example, conifer forests were found to withstand drought impacts better than broadleaf forests in Canada (Kljun et al., 2006) and in Europe during the extremely dry year of 2003 (Granier et al., 2007). However, the present generation of ecosystem models embody a simplistic representation of drought responses of these ecophysiological properties and processes across forest ecosystems, which makes it difficult to predict the likely extent of drought-induced changes in the function of forest ecosystems.

Current state-of-the-art Earth System models (ESMs), which include dynamic global vegetation models (DGVMs) (Prentice and Cowling, 2013) coupled to physical representations of land-atmosphere exchanges of energy, water vapor and CO_2_ (land surface models, LSMs), make widely divergent predictions of drought effects (Prentice et al., 2015). This divergence is partly due to the lack of an established, empirically supported method for the representation of drought effects on plants (Egea et al., 2011). Process-based modeling of the drought impacts on plants and ecosystems must be informed by experiments, which can help us to understand underlying processes. Model evaluation and improvement must include the use of experimental observations, theory to explain the observations, quantitative parameterizations to describe the theory, and model simulations to test the impacts of environmental variables (Bonan et al., 2014; Prentice et al., 2015; see a model-data integration process by Walker et al., 2014). Although thousands of experiments have been done to study the drought responses of plants, relatively few provide information in the quantitative manner required to develop model representations. In particular, work is needed (1) to directly determine how different aspects of plant function respond to experimentally imposed drought and (2) to analyze experimental results in a theoretical framework, suitable for inclusion in ecosystem models.

In particular, current ecosystem models differ greatly in the ways in which they represent drought effects on photosynthesis (Medlyn et al., 2016). Many models simulate the drought effect on photosynthesis in a rough way simply by reducing the slope of the relationship between stomatal conductance (*g*_s_) and net carbon assimilation rate (*A*_n_) (Egea et al., 2011), in a similar way for all plant function types (PFTs). Another specific issue is that the apparent maximum carboxylation rate (*V*_cmax_) has usually been attributed to a PFT as a single value, or as a single-valued function of environmental drivers (Haxeltine and Prentice, 1996). It is not known whether the method is adequate to capture the drought response, but there is a strong case to expect that it is not, and it does not account for either differences among species and/or ecosystems of different climatic origins, or for mechanisms of plant acclimation to drought. Emerging modeling evidence points to the importance of representing both stomatal and non-stomatal responses to drought in models (e.g., Egea et al., 2011; De Kauwe et al., 2015b). However, the modeling approach in current LSMs lacks a functionally realistic representation of drought responses of *g*_s_ and *V*_cmax_, unless the experimentally based and PFT-specific representations of the drought responses of *g*_s_ and *V*_cmax_ have been implemented.

Recently, there are increasingly more model-experiment synthesis studies to improve the representation of photosynthetic responses to environmental drivers in large-scale vegetation models (e.g., Medlyn et al., 2011; Prentice et al., 2014; De Kauwe et al., 2015a,b). In this review, we highlight recent studies which analyze the experimental data of both the short- and long-term drought responses of leaf gas exchange across species of contrasting climatic origins, and which aim at improving the representation of experimentally based and PFT based stomatal and non-stomatal response functions to soil water stress in LSMs and DGVMs.

## Which Photosynthetic Processes Are Affected Under Short-Term Drought?

**FIGURE 1:**
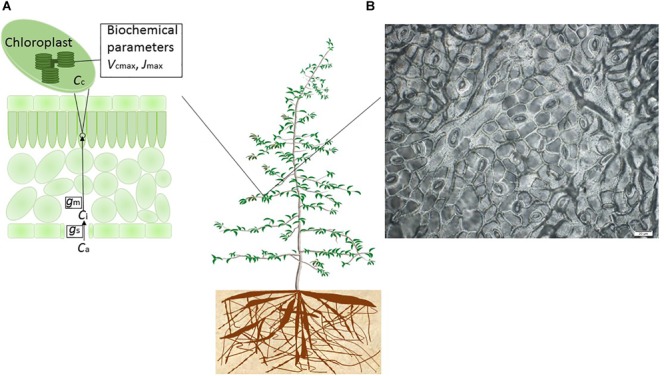
Conceptual diagram of the model-experiment synthesis framework to quantify and model the differential sensitivities of leaf gas exchange to drought. **(A)** During photosynthesis, the CO_2_ flux from the air (ambient CO_2_ concentration, *C*_a_) to intercellular air spaces (intercellular CO_2_ concentration, *C*_i_) through stomata is limited by stomatal conductance (*g*_s_). The CO_2_ flux from the intercellular space to the chloroplast site (CO_2_ concentration at the chloroplast, *C*_c_) is limited by mesophyll conductance (*g*_m_). The non-stomatal limitation on photosynthesis was attributed to drought effects on *g*_m_, the actual maximum rate of CO_2_ consumption by RuBP carboxylation by Rubisco (*V*_cmax_) and the actual maximum electron transport rate (*J*_max_). **(B)** An impression of the leaf epidermis of *E. camaldulensis* subsp. *camaldulensis* was produced using clear nail polish, which then was mounted for applications of microphotography of the leaf surface. The microscope (Olympus BX53, Olympus America Inc.) was interfaced with a digital camera at ×40 magnification. Photo: Shuang-Xi Zhou.

Leaf *A*_n_ is mainly driven by light, temperature and intercellular CO_2_, as represented in the Farquhar-von Caemmerer-Berry leaf-level photosynthesis model for C_3_ plants (Farquhar et al., 1980). Intercellular CO_2_, in turn, is co-determined by *g*_s_ and *A*_n_. Reduction of *g*_s_ is one of the foremost, short-term, leaf-scale physiological responses both to atmospheric vapor pressure deficit (the driving force of transpiration, *E*) and soil water deficit. CO_2_ and water vapor exchange are strongly coupled through stomata, because *g*_s_ regulates both the CO_2_ uptake for photosynthesis, and the loss of water vapor by transpiration (Cowan, 1977) (Figure 1). A variant of the Farquhar-von Caemmerer-Berry model was coupled to the empirical Ball–Berry stomatal conductance model (Ball et al., 1987; Collatz et al., 1991) in the land component of climate models already in the mid-1990s, in order to estimate gross primary production on a more mechanistic basis than before (Bonan, 1995; Cox et al., 1998). This or other similar formulations are now used widely in state-of-the-art ESMs. Research efforts have also been devoted specifically to the implementation of different modeling approaches for stomatal conductance (e.g., Bonan et al., 2014; De Kauwe et al., 2015a).

Although photosynthesis accounts for the largest CO_2_ flux from the atmosphere into ecosystems and is the driving process for terrestrial ecosystem function (Bernacchi et al., 2013), the fundamental component processes of plant gas exchange are still incompletely represented in global models, notably in the area of drought responses, and photosynthetic and morphological acclimation generally (including acclimation to drought) (Prentice and Cowling, 2013). In ecosystem models, drought stress may act either by increasing the marginal water use efficiency, which depends on the ratio of CO_2_ concentration inside and outside the leaf (*C*_i_/*C*_a_ ratio); by reducing *V*_cmax_ and/or the maximum rate of electron transport – *J*_max_ (apparent values implicitly assuming infinite *g*_m_); or both (Figure 1). Accurate model prediction of drought impacts on vegetation and global carbon and water cycles requires realistic representation of photosynthetic processes at the leaf level (Baldocchi, 1997; Egea et al., 2011; Verhoef and Egea, 2014).

Stomatal behavior is expected to be related to the marginal carbon cost of water loss (*λ* = ∂A/∂E) (Cowan, 1977; Cowan and Farquhar, 1977; Berninger and Hari, 1993). Cowan and Farquhar (1977) postulated that for any given amount of total water available for transpiration in a period of time, the leaf can achieve the maximum CO_2_ uptake if it adjusts leaf scale conductance in the way that the derivative of *A*_n_ with respect to the rate of transpiration per unit area of leaf (∂A/∂E) is maintained constant throughout the period (Cowan, 1977). This criterion amounts to saying that a plant with a given water availability regulates stomata to ensure maximal carbon gain per unit water loss in a finite period of time. Therefore, the constancy of ∂A/∂E is viewed as an optimality hypothesis (Cowan, 1977; Cowan and Farquhar, 1977). It has also been suggested that the rate at which water stress is imposed might influence the response of ∂A/∂E to water stress (Hall and Schulze, 1980). The theoretical analysis of Mäkelä et al. (1996) further predicted that the marginal water cost of carbon (1/*λ*) should decline exponentially with decreased soil moisture, and that the rate of decline should increase according to the probability of rain.

Medlyn et al. (2011) and Prentice et al. (2014) have proposed re-interpretations of widely used empirical models of stomatal conductance, in terms of optimization theory. Medlyn et al. (2011) derived a simple expression that is a good approximate solution of the Cowan-Farquhar optimization problem, and demonstrated its predictive power for a range of species. The single parameter of the Medlyn et al. (2011) optimal model for stomatal behavior – the stomatal sensitivity parameter (*g*_1_) – is inversely proportional to *λaa*, and thus can be used directly to test the predictions by Mäkelä et al. (1996) (Héroult et al., 2013; see a conceptual modeling framework in Zhou et al., 2013). Prentice et al. (2014) introduced a different derivation of the same expression, with further empirical support, based on the alternative hypothesis that plants minimize the sum of the unit costs (carbon expended per unit assimilation) of CO_2_ uptake and water loss. Different expressions again have been presented by Sperry et al. (2016), Wolf et al. (2016), and Dewar et al. (2018) based on the optimization criterion that plants maximize carbon gain by minimizing carbon costs associated with hydraulic failure.

Besides the stomatal resistance on CO_2_ diffusion from the atmosphere to the intercellular air spaces of the leaves, there is now known to be a considerable mesophyll resistance to CO_2_ diffusion from the substomatal cavity to the carboxylation sites in the chloroplasts (Figure 1). In other words, there is a mesophyll conductance (*g*_m_) which is not infinite and can significantly limit the CO_2_ availability and thus the assimilation rate. *g*_m_ has been shown to play an important role in determining photosynthetic responses to environmental drivers including temperature and CO_2_ (e.g., Niinemets et al., 2011; Evans and von Caemmerer, 2013). Photosynthesis is reported to be limited by decreased *g*_m_ – together with *g*_s_ – in the initial stages of drought (Bota et al., 2004; Flexas et al., 2004, 2007, 2008, 2012; Grassi and Magnani, 2005; Egea et al., 2011; Zhou et al., 2014). There has been controversy on the magnitude of the *g*_m_ effect on photosynthesis under mild to moderate drought conditions, largely due to the methodological issues on estimation of the intercellular or the chloroplastic CO_2_ concentration (*C*_c_; Figure 1) (Pinheiro and Chaves, 2011). In addition, there is controversy on whether *g*_m_ should be included in ecosystem models, and how to include *g*_m_ in ecosystem models (Rogers et al., 2017). Some recent studies suggested that the decrease of *g*_m_ with increasing soil water deficit could contribute as much as the decrease of *g*_s_ to the reduction of *A*_n_ under drought (e.g., Flexas et al., 2012; Zhou et al., 2014). However, far less is known on the environmental regulation and interspecific differences in *g*_m_ compared to *g*_s_.

As plant water status worsens, there is a further possibility that drought impedes enzyme activity and photosynthetic capacity. In other words, there can be directly drought-induced biochemical limitations on the activity of Rubisco (ribulose-1,5-bisphosphate carboxylase/oxygenase) and the regeneration capacity of RuBP (ribulose-1,5-bisphosphate) (Kanechi et al., 1996; Tezara et al., 1999; Castrillo et al., 2001; Parry et al., 2002; Tezara et al., 2002; Thimmanaik et al., 2002; Grassi and Magnani, 2005). Drought-induced decrease of Rubisco activity is associated with down-regulation of the activation state of the enzyme (e.g., by de-carbamylation and/or binding of inhibitory sugar phosphates). In the Farquhar-von Caemmerer-Berry model (Farquhar et al., 1980), the *V*_cmax_ and *J*_max_ (apparent values implicitly assuming infinite *g*_m_) are the two key metabolic parameters limiting photosynthetic capacity (Figure 1). Varying among leaves within a plant, between plants, among species and seasonally (e.g., Wilson et al., 2000; Xu and Baldocchi, 2003), *V*_cmax_ plays an important role in linking the carbon fluxes between the leaves and the atmosphere and thus in governing plant productivity and resource use efficiency (Long et al., 2006) and determining large-scale fluxes of CO_2_ between vegetation and the atmosphere (Bonan et al., 2011, 2012). The trigger for decreased Rubisco activity is reported to depend on the severity and/or the duration of the stress imposed (Flexas et al., 2006). Ecosystem models commonly assign fixed values of *V*_cmax_ per PFT, but there is no consistency in the values assigned among different models and, in any case, this approach neglects most of the field-observed variation in *V*_cmax_ (Kattge et al., 2009; Bonan et al., 2011, 2012; Groenendijk et al., 2011). Recent studies have suggested that it is necessary to represent the effects of climate change on *V*_cmax_ in models to predict its impact on net primary production (e.g., Bernacchi et al., 2013; Galmés et al., 2013).

It is generally thought that with the increase of drought intensity and/or duration, biochemical limitations on photosynthesis should eventually come to dominate over diffusional (stomatal and mesophyll) limitations (see a review by Lawlor and Tezara, 2009). However, there has been a good deal of debate about the relative importance of photosynthetic limitations of diffusive and biochemical origin, in the context of drought (e.g., Grassi and Magnani, 2005). Reasons for controversy include the use of different measures of drought, the imposition of drought at different rates in experiments, different applied intensities and duration of drought, different experimental designs and growth conditions, and different species with different physiological and structural sensitivities and adaptations to drought.

## Interspecific Variation in the Short-Term Stomatal and Non-Stomatal Drought Responses Among Species From Different Hydro-Climates

The drought responses of different species are likely to depend not only on drought duration and intensity, but also on the species-specific degree of adaptation to the soil water conditions in their native habitat. It is well documented that plants from dry climates can operate better than plants from wet climates down to severe soil water deficits (e.g., Sperry, 2000). However, studies have highlighted that mesic and xeric forest ecosystems are equally vulnerable to drought-induced mortality, based on their functional hydraulic limits (Choat et al., 2012), implying that plants from drier or wetter environments possess some degree of adaptation to the soil conditions encountered in their native habitat. Indeed, xeric species were reported to keep stomata open and maintain photosynthesis down to lower water potential values than mesic species (e.g., Zhou et al., 2014). It is reasonable to assume that this feature of plants from dry climates is adaptive, important for their function under field conditions and shaping their potential geographic ranges (Engelbrecht et al., 2007). Differential drought adaptations among species are presumed to underpin their different levels of sensitivity, resistance, and resilience to soil water deficits (Chaves et al., 2003; McDowell et al., 2008), and differential effectiveness of physiological mechanisms of drought tolerance in the face of decreasing water potential (Engelbrecht et al., 2007). The wide variation of drought adaptations among species is likely to be fundamentally important in determining their different degrees of vulnerability to biomass loss and mortality (Ciais et al., 2005; Adams et al., 2009; Allen et al., 2010).

Under a drier and hotter climate, the intra- and inter-specific variation in plant traits may provide an important contribution to plants’ resistance to drought, with responses characteristic of plants from dry environments promoting persistence and adaptation, reducing risk of mortality and improving chance of survival (Yachi and Loreau, 1999; Clark et al., 2012). Understanding how these effects vary among species from contrasting climates is key to predicting the large-scale consequences of drought on different communities and ecosystems. Very few published experiments have systematically tested how the various components of plant drought response vary across species from contrasting hydro-climates. The response of the stomatal sensitivity parameter (*g*_1_) of the Medlyn stomatal optimality model (Medlyn et al., 2011) to soil water deficit is expected *a priori* to differ among plant functional types and species of different geographical origins (Medlyn et al., 2011; Héroult et al., 2013). Meanwhile, *V*_cmax_ is expected to be higher in plant species from drier climates (Prentice et al., 2014), in compensation for reduced stomatal conductance. There is also significant variability in the Rubisco specificity factor among closely related C_3_ higher plants, which is associated mainly with temperature and water availability (Galmés et al., 2005). Zhou et al. (2013, 2014) fitted quantitative models for species of different origin of climate and PFT membership in their differential *g*_s_ and *V*_cmax_ responses to soil water potential – providing functions that potentially could represent these responses in process-based models.

**FIGURE 2:**
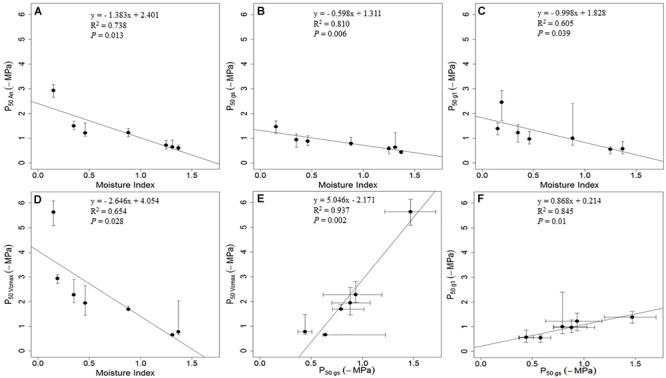
Correlation between the pre-dawn leaf water potential at 50% loss of photosynthetic functions and moisture index for six woody species from contrasting hydroclimates. **(A)** The pre-dawn leaf water potential at 50% loss of net carbon assimilation rate (P_50An_, -MPa) and moisture index. **(B)** The pre-dawn leaf water potential at 50% loss of stomatal conductance (P_50gs_, -MPa) and moisture index. **(C)** The pre-dawn leaf water potential at 50% loss of stomatal sensitivity parameter *g*_1_ (P_50g1_, -MPa) and moisture index. **(D)** The pre-dawn leaf water potential at 50% loss of RuBP carboxylation by Rubisco (P_50Vcmax_, -MPa) and moisture index. **(E)** Correlation between P_50Vcmax_ and P_50gs_. **(F)** Correlation between P_50g1_ and P_50gs_. Moisture index is the ratio between mean annual precipitation and mean annual potential evapotranspiration, which can range from zero in the driest regions to higher values in wetter regions (Zhou et al., 2014). Values of P_50An_, P_50gs_, P_50g1_ and P_50Vcmax_ (solid circle) – and the bootstrap 2.5% and bootstrap 95% values (bars) to indicate the error in each estimate – were fitted by employing data from Zhou et al. (2014) using the ‘fitplc’ package in R (Duursma and Choat, 2017).

The decline rates of stomatal and hydraulic function with decreasing water potential levels were reported to be coordinated across species of different climate of origin (Choat et al., 2012; Klein, 2014; Manzoni, 2014; Martin-StPaul et al., 2017; Li et al., 2018; but see Lamy et al., 2014). In this review, we calculated the pre-dawn leaf water potential at which 50% loss of full functions occurred (P_50_, -MPa) for the photosynthetic parameters reported in Zhou et al. (2014) – 50% loss of net carbon assimilation rate (P_50An_, -MPa), 50% loss of stomatal conductance (P_50gs_, -MPa), 50% loss of stomatal sensitivity parameter *g*_1_ (P_50g1_, -MPa) and 50% loss of RuBP carboxylation by Rubisco (P_50Vcmax_, -MPa) (Figure 2). We found P_50_ was more negative for *A*_n_ than for *g*_s_, indicating that the reduction in stomatal conductance preceded the reduction in photosynthesis (Figures 2A,B). When comparing how stomatal sensitivity parameter *g*_1_ and *V*_cmax_ contributed to the overall reduction in *g*_s_, we found P_50g1_ and P_50Vcmax_ were correlated, while the P_50Vcmax_ was consistently more negative than P_50g1_ – indicating the reduction in *g*_1_ preceded the reduction in *V*_cmax_ (Figures 2E,F). Moreover, compared with species from wetter climates, species from drier climates tends to show larger difference between P_50Vcmax_ and P_50gs_ (Figure 2). These differences are presumed to have adaptive significance for the survival of plants in dry climates.

## Interspecific Variation in the Degree of Plant Photosynthetic Acclimation to Prolonged Water Stress

Plants subjected to short-term experimental drought are well documented to experience a decline in photosynthetic capacity. However, plants under prolonged drought may be able to acclimate to drought to some extent, for example through morphological adjustments such as changes in mass allocation to leaves and/or roots (Choat et al., 2018), provided the drought is imposed slowly enough for such changes to take effect. In general, therefore, it is to be expected that the mechanisms underlying plant responses to water stress vary according to time scale (Maseda and Fernández, 2006; Limousin et al., 2010a,b; Martin-StPaul et al., 2012, 2013). Maseda and Fernández (2006) proposed that plants acclimate to drought at the whole-organism level through physiological, anatomical, and morphological adjustments that are adaptive over a time scale of months.

**FIGURE 3:**
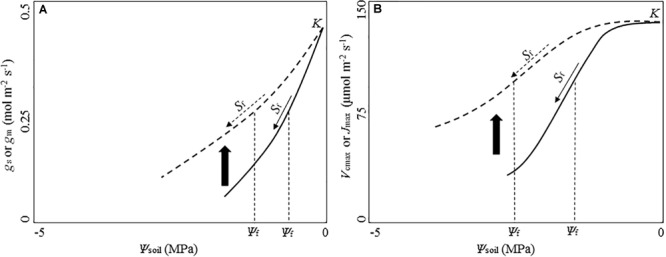
Conceptual diagram of potential acclimation responses of diffusional and biochemical parameters to water stress. Solid lines show the strong short-term sensitivity of parameters to the decreasing soil water potential (*Ψ*_soil_) before plants being given time to acclimate to water stress. Dashed lines indicate reduced sensitivity to soil water potential following a period of acclimation to water stress. Upward arrow between the solid and dashed line shows potential shift upward of the drought-response curve during acclimation. **(A)** Exponential function of stomatal conductance (*g*_s_) and mesophyll conductance (*g*_m_) with the decreasing *Ψ*_soil_ based on Zhou et al. (2013, 2014). **(B)** Logistic function of the maximum rate of CO_2_ consumption by RuBP carboxylation by Rubisco (*V*_cmax_) and the maximum electron transport rate (*J*_max_) with the decreasing *Ψ*_soil_ based on Zhou et al. (2013, 2014). *K* is the maximum value of the parameter under moist conditions. *S*_f_ indicates the sensitivity of each parameter against the decreasing *Ψ*_soil_. *Ψ*_f_ is soil water potential at 50% reduction of *K*.

When given time to acclimate to water stress, the photosynthetic response of plants could differ from that of plants in short-term water stress (Figure 3). Some longer-term experiments reported higher leaf gas exchange rates for woody plants in the drought treatment (Cinnirella et al., 2002; Ogaya and Peñuelas, 2003; Llorens et al., 2004). Cano et al. (2014) reported xeric species showed significant higher *g*_m_ than mesic species under longer-term water stress. Leaves developed during the long-term drought can acclimate by increasing partitioning to total soluble proteins, allowing higher Rubisco activity per unit leaf area (Panković et al., 1999). The Rubisco content could also increase in leaves under prolonged drought, and the increase could be significantly higher in leaves of the drought-tolerant plant taxa than other taxa, conferring the drought-tolerant taxa with better acclimation and higher drought tolerance (Panković et al., 1999).

Despite its importance, the photosynthetic responses of plants to long-term water stress and its variation among species of contrasting climate of origin are poorly understood (Cano et al., 2014). Moreover, long-term studies disagree on whether or not plants can modify the functional relationships between photosynthetic traits and soil water potential to acclimate to long-term water stress (Limousin et al., 2010b; Misson et al., 2010; Martin-StPaul et al., 2012). There could be systematic differences in these functional relationships related to species’ climatic origin. Zhou et al. (2016) found the xeric *Eucalyptus* species showed more effective drought acclimation – significantly lower *V*_cmax_ sensitivity to declined pre-dawn leaf water potential – than the riparian species under prolonged drought. Species-specific physiology may play an importance role in the comparative photosynthetic acclimation of contrasting species under prolonged water stress, leading to the varied findings among these studies (Ogaya and Peñuelas, 2003; Cano et al., 2014).

Intra- and inter- specific variation in drought tolerance and acclimation could have important implications for forest modeling in water-limited ecosystems, particularly in a long-term perspective that takes future climate change into account. Ignoring potentially important acclimation processes in the field could lead to overestimation of the long-term consequences of drought. Changes in forest composition related to drought tolerance and acclimation are already beginning to be observed. For example, the more drought-tolerant *Quercus pubescens* was reported to be replacing *Pinus sylvestris* at low altitudes in Switzerland, where climate change has brought about recurrent water deficits (Eilmann et al., 2006). Reliable prediction of drought effects on contrasting species and forest ecosystems under field conditions requires long-term experiments on the drought-induced limitations on photosynthetic and hydraulic properties, and their potential acclimation to prolonged drought (see a review by Choat et al., 2018). The number of such studies in the literature, however, is surprisingly small, with most published manipulative experiments focusing exclusively on short-term responses to drought.

## Incorporating Experiment-Derived Pft-Specific Drought Response of *g*_s_ and *V*_cmax_ to Improve Modeling Ecosystem Responses to Drought

Current LSMs treat plant ecophysiological properties simplistically assuming the same drought sensitivity for all vegetation (Prentice and Cowling, 2013; De Kauwe et al., 2015b), disregarding known aspects of trait correlation and trait-environment relationships (Wright et al., 2004; Maire et al., 2013; Prentice et al., 2014) and the considerable variation of drought sensitivity among plant species of different climatic origin highlighted in recent model-oriented experiments and data syntheses (Zhou et al., 2013, 2014). Insufficient attention has been paid to the evaluation of LSMs in their representations of the fundamental eco-physiological responses to drought, in part because their early history of development pre-dates the availability of many relevant measurement data sets (Prentice et al., 2015). It is critical for LSMs to realistically represent the differential drought responses of different vegetation types.

Largely due to the shortage of model-oriented experimental studies describing the separate effects of drought on stomatal and non-stomatal processes, there are large discrepancies in the ways in which current ecosystem models represent the drought effect on plant gas exchange (Powell et al., 2013; Medlyn et al., 2016). There has been a scientific debate on how to represent stomatal closure as soil moisture declines (Bonan et al., 2014). Current state-of-the-art LSMs used in coupled climate models generally treat all PFTs as experiencing similar stomatal and/or non-stomatal limitation during drought (via soil texture and assumed rooting depths). Many LSMs use an empirical soil moisture stress factor (β) – as a function of volumetric water content (𝜃) – to impose for down-regulation of stomatal response at decreasing soil moisture, which allowing an abrupt transition in β to take place within a narrow range of 𝜃 (Egea et al., 2011; Powell et al., 2013; De Kauwe et al., 2015a,b; but see Medlyn et al., 2016). Powell et al. (2013) reported unrealistic drought responses due to implementing abrupt transitions of this kind in four models [Community Land Model version 3.5 (CLM3.5), Integrated BIosphere Simulator version 2.6.4 (IBIS), Joint UK Land Environment Simulator version 2.1 (JULES), and Simple Biosphere model version 3 (SiB3)], which use different water-stress functions – loosely constrained by data – to down-regulate soil moisture effects on *g*_s_. The sharp shutdown seems to be a common challenge in LSMs whereas the observed fluxes decline much more gradually with water stress (Medlyn et al., 2016). Zhou (2015) compared the 𝜃 effect on *A*_n_ in the Community Atmosphere Biosphere Land Exchange (CABLE) LSM, and found a rather abrupt transition in *A*_n_ from near-normal function to nearly complete shutdown within a narrow range of 𝜃 – regardless of PFT or soil type. This modeled abrupt transition is very unlikely under field conditions where the transition from full vegetation function to drought conditions should occur more gradually as a consequence of spatial heterogeneity in plant and soil properties (Liang et al., 1994; Prentice et al., 2015).

Land surface models commonly include generic responses of plant carbon uptake and water loss to soil moisture content. It seems plausible that the performance of LSMs might be improved by including empirically based plant responses to drought, expressed as a function of soil water potential (the key property affecting plant water uptake) and derived from measurements on species of different PFT membership. However, the process of estimation of required model parameters (e.g., quantifying the response functions of photosynthetic and hydraulic traits against drought) for global models is not straightforward and usually not transparent (Choat et al., 2018; Dewar et al., 2018). De Kauwe et al. (2015b) tested whether using the information pertinent to the representation of *g*_s_ and *V*_cmax_ responses in process-based models in Zhou et al. (2013, 2014) would improve the prediction of canopy-atmosphere fluxes during drought in the CABLE model. By estimating soil water potential from dynamically weighted soil layers, De Kauwe et al. (2015b) resolved the modeling challenge in CABLE – the steep drop-off of leaf photosynthesis with soil water content due to the rapid change in soil moisture potential (Zhou, 2015). It is found that CABLE can only accurately reproduce the drought impacts during the 2003 heat wave if the most mesic sites were attributed a high drought sensitivity and the most xeric sites were attributed a lower drought sensitivity (De Kauwe et al., 2015b). These studies demonstrated a practical and effective approach to gain information on drought responses in a form directly applicable to modeling, and highlighted that LSMs will over-estimate the drought impacts in drier climates if the different sensitivity of vegetation to drought were not taken into account (De Kauwe et al., 2015b; Zhou, 2015).

Furthermore, recent efforts to improve model simulation on vegetation dynamics also have highlighted the importance of linking plant traits – especially the correlation among hydraulic and photosynthetic traits (e.g., the water potentials at 50% loss of xylem conductivity and 50% loss of stomatal conductance, respectively) – to forest function under drought (Christoffersen et al., 2016; Xu et al., 2016; see a review by Choat et al., 2018). Christoffersen et al. (2016) represented the correlations between plant hydraulic traits and the leaf and stem economic traits within a trait-driven model, and found substantial improvement of the model simulations of total ecosystem transpiration fluxes. Xu et al. (2016) updated the Ecosystem Demography model 2 with a novel hydraulics-driven phenology scheme, which incorporated PFT-specific functional traits and allowed alternative photosynthetic and phenological strategies to dominate depending on rainfall seasonality, and found it substantially improved the model simulation of spatiotemporal patterns of vegetation dynamics in seasonally dry tropical forests.

## Significance of Model-Experiment Synthesis: Future Perspectives

Increasing research efforts are devoted to improving models through the use of new representations of specific processes based on new data syntheses or experimental findings (e.g., Bonan et al., 2011, 2012; Medlyn et al., 2011, 2016; Prentice et al., 2014; De Kauwe et al., 2015a,b; Christoffersen et al., 2016; Xu et al., 2016; Rogers et al., 2017; see a model-data integration process by Walker et al., 2014). These studies have highlighted the significance of bridging experiment-tested eco-physiological processes and land ecosystem models, through translating empirical findings into improved process representations within models and tested model simulations against carbon and water flux measurements at the ecosystem scale.

Both plant physiologists and modelers should be exposed to the significance of informing process-based models with experimentally derived quantitative information when studying the drought impacts on photosynthetic and hydraulic properties, their variation across forest ecosystems and their interaction underlying short-term and prolonged drought consequences. The model-orientated experimental work would ideally be carried out to identify different drought tolerances in a quantitative modeling context – both in respect of short-term drought responses, and acclimation processes by which plants can adapt to longer-term, lower-level drought – between species from mesic and xeric habitats.

In this review, the new analysis highlights that the drought sensitivities of photosynthesis are consistently higher for species from wetter climates (showing strong diffusional and metabolic limitations earlier during the drying-down process) than species from drier climates (showing more negative pre-dawn leaf water potential at 50% reduction of diffusional and metabolic activities) (Figure 2). The positive correlations among the rates of decline of the parameters as the experimental drought progressed define a spectrum of drought adaptations, from more resistant species thriving in dry environments, to more sensitive species thriving in moist environments (Figure 2; but see Lamy et al., 2014). These findings support the existence of a co-ordinated spectrum of increasing tolerance in plants from wetter to drier environments, and provide a complementary perspective on the finding by Choat et al. (2012) based on hydraulic traits that trees in mesic habitats – which are not normally considered to be at risk from drought – are actually just as vulnerable to drought as trees in xeric habitats. Meanwhile, these studies have casted light on the general responses of leaf gas exchange to short- and long-term soil water deficits, allowing for realistic model representations of different drought sensitivities among species and PFTs from wetter or drier environments. By providing process-based analytical models of key parameters that define these responses for contrasting species with differential drought sensitivities, these model-oriented experimental studies have offered potentially robust solutions to the problem of representing adaptive differences among PFTs into LSMs.

This review suggests the following priorities to help guide future research to improve modeling on drought compacts upon a firm theoretical and empirical basis:

(1) Plants are subject to Darwinian selection and can adjust genetically or phenotypically to environment to maximize their fitness. Optimality concepts have been proposed and tested on various aspects of plant and ecosystem functions including plant water use, stomatal behavior, photosynthetic capacity, nitrogen uptake and phenology (e.g., Prentice et al., 2014). Covariation of different plant traits is expected to be an expression of optimality principles (Wright et al., 2004; Prentice et al., 2014; Reich, 2014) and should simplify the parameterizations of fundamental eco-physiological responses to environmental drivers, including drought. An explicit theoretical framework is necessary to incorporate the variation, interrelationships and environmental dependencies of plant traits into models. Such quantitative information explaining fundamental plant-level processes and quantifying variability in key plant traits under drought, if gathered on a wider range of species, could allow testing for the existence of a spectrum of drought-response traits, and ultimately a deeper understanding of drought-response strategies (Wright et al., 2004; Prentice et al., 2014; Reich, 2014) and a more comprehensive approach to both trait data analysis and vegetation modeling (Prentice et al., 2014).
(2) Model predictions of future drought impacts on ecosystems, and feedbacks to the atmosphere, should aim to represent drought responses of major plant physiological exchanges (CO_2_ and water vapor fluxes between leaf and the atmosphere) realistically, with simple and observationally tested process formulations. They must account for the observed differences between the responses of different PFTs to drought. The realistic model representation of stomatal and non-stomatal responses to short-term and prolonged drought – and their variation among plants of different PFT membership and/or climatic origin – will be fundamental to the prediction of drought-induced mortality at plant scale (due to hydraulic failure, carbon starvation, and/or other mechanisms), or carbon loss at the ecosystem scale. Varied vegetation sensitivity to drought are necessary for LSMs to accurately explain the large-scale patterns of drought response of carbon, water and energy fluxes observed in different environments (De Kauwe et al., 2015b).
(3) Time scale may be of the essence when determining the extent to which climate change is likely to adversely affect forests. Drought acclimation is evidently a real phenomenon in trees adapted to dry climates, and presumably allows such trees to cope with periodic, protracted (but not too severe) droughts. The inherent differences among the species from contrasting climatic origins can be shown not only in their contrasting degree of tolerance to short-term drought (e.g., Zhou et al., 2013, 2014), but also in their contrasting abilities to compensate for long-term drought (e.g., Zhou et al., 2016). Drought-induced mortality of trees, and carbon loss from forests, could be overestimated if such acclimation is not taken into account (Choat et al., 2018). Model projections of drought effects on species distributions and vegetation composition in climate change scenarios should consider the differences in both short-term drought sensitivity and longer-term acclimation potential among species adapted to different climates.

The studies in this review mainly consider short-term dynamics of stomatal behavior and marginal water use efficiency, whose temporal dynamics could differ across seasons and years (Chen et al., 2018). Incorporation of their long-term dynamic patterns – such as the potential difference between dry versus wet seasons and across forest ecosystems – into ecosystem models remains to be improved. Besides, future model-experiment inter-comparison analysis needs to improve the representation of photosynthetic responses to co-occurring environmental stresses, such as drought and heat wave – which usually occur together (Ciais et al., 2005; Granier et al., 2007). High temperature can cause severe impacts on the photosynthetic apparatus, particularly during long-lasting drought events. Meanwhile, forest ecosystems at hot and dry environments – where stomatal closure (contributing to higher leaf temperature) and low *C*_c_ are necessary for plants to conserve water and avoid hydraulic failure (see a review by Choat et al., 2018) – could show very different responses of Rubisco characteristics and photosynthetic capacity under drought and high temperature conditions (Delgado et al., 1995; Kent and Tomany, 1995; Galmés et al., 2005). The model-experiment synthesis work in this area will enhance our predictions of environmental change consequences on forestry ecosystems.

## Conclusion

Investigating the general trends of trait variation with key environmental factors, and translating this variation into improved process representation in vegetation models, are important developments for the improvement of LSMs and DGVMs. The model-oriented data analysis, experiments, and modeling described in this review amount to a new synthesis of information on the responses of different plant functions to drought, and provide a general methodology for systematic study of the relationship between plant processes and drought, allowing the derivation of functions that can be used directly in modeling. They can be seen as part of a wider movement toward the observationally driven parameterization of fundamental vegetation processes. Such work can also contribute to climate-change adaptation, through facilitating more accurate predictions of how forestry systems are likely to respond to projected changes in drought intensity and duration in a rapidly changing world.

## Author Contributions

S-XZ drafted the work. All authors contributed substantially to the conception of this work and critically revised the work. ICP secured the funding.

## Conflict of Interest Statement

The authors declare that the research was conducted in the absence of any commercial or financial relationships that could be construed as a potential conflict of interest.
